# Clinical risk factors and machine learning for venous thromboembolism in lung cancer: an exploratory single-center study

**DOI:** 10.3389/fmed.2026.1891648

**Published:** 2026-07-31

**Authors:** Hongqin Jia, Yu Chen, Kang Qian, Hongbin Zhu

**Affiliations:** 1 The Fourth Affiliated Hospital of Anhui Medical University, Chaohu, Anhui, China; 2 Department of Respiratory and Critical Care Medicine, The Fourth Affiliated Hospital of Anhui Medical University, Chaohu, Anhui, China

**Keywords:** chemotherapy, D-dimer, lung cancer, machine learning, venous thromboembolism

## Abstract

**Background:**

Venous thromboembolism (VTE) represents a common and severe comorbidity in lung cancer patients. This study aimed to characterize the clinical features and independent risk factors associated with VTE in these individuals, and to evaluate the complementary utility of machine learning approaches in enhancing VTE risk stratification.

**Methods:**

This retrospective study included lung cancer patients admitted to the Fourth Affiliated Hospital of Anhui Medical University between January 2018 and April 2025. According to imaging findings, patients were categorized into a VTE group and a control group. Clinical characteristics, laboratory parameters, and treatment-related information were retrospectively collected and analyzed. Univariate and multivariable logistic regression analyses were performed to identify factors independently associated with VTE. Additionally, four machine learning models—Ridge-LR, LASSO, random forest (RF), and extreme gradient boosting (XGBoost)—were developed for exploratory purposes. Model performance was evaluated using the area under the receiver operating characteristic curve (AUC), sensitivity, specificity, calibration metrics (Brier score, calibration slope and intercept, Hosmer-Lemeshow test), and decision curve analysis (DCA).

**Results:**

A total of 180 patients with lung cancer were included. Elevated D-dimer levels were independently associated with increased VTE risk (OR = 1.084, *p* = 0.021). Chemotherapy exposure showed an OR of 2.858 (95% CI: 1.081–7.555, *p* = 0.034) for VTE in the 1–2 cycle subgroup, although no significant association was observed for higher cycle numbers, suggesting potential confounding. Male sex was independently associated with embolic events occurring outside the lower extremities (OR = 10.83, *p* = 0.034). Among the machine learning models, RF achieved the highest AUC (0.740), but Ridge-LR demonstrated the best calibration (Hosmer-Lemeshow *p* = 0.345). LASSO showed the highest net benefit on decision curve analysis, while RF and XGBoost exhibited significant miscalibration (*p* = 0.002 for both). D-dimer (41.3%) and total protein (34.7%) were identified as the most important predictors in the XGBoost model.

**Conclusion:**

Elevated D-dimer levels measured prior to VTE diagnosis during routine clinical monitoring were independently associated with subsequent VTE, supporting its potential utility as a predictive biomarker for risk stratification in patients with lung cancer. The association between chemotherapy exposure and VTE is likely influenced by selection bias and residual confounding rather than representing a true causal effect. Machine learning models did not demonstrate consistent improvement over logistic regression in discrimination or calibration in this small single-center cohort, suggesting limited added predictive value with the current dataset.

## 1 Introduction

Venous thromboembolism (VTE), primarily comprising deep vein thrombosis (DVT), pulmonary embolism (PE), and splanchnic vein thrombosis (SVT) (1, 2), represents a significant global health burden. The annual incidence ranges from 39 to 115 per 100,000 individuals for PE and 53–162 per 100,000 for DVT. Acute PE ranks as the third most common acute cardiovascular syndrome worldwide, following myocardial infarction and stroke (3, 4).

Lung cancer is one of the most frequently diagnosed cancers and ranks among the leading causes of cancer-related mortality globally, with a 5-year survival rate of only 1020% (5). It is consistently identified as one of the cancer types conferring the highest risk for VTE and is considered a predisposing factor for predicting VTE recurrence risk (3, 4). Cancer patients exhibit an increased risk of both venous and arterial thromboembolism (VTE/ATE) (6). Specifically, the risk of VTE is 5–6 times higher in cancer patients compared to those without cancer (7, 8). Among lung cancer patients, the incidence of VTE is notably high, reaching up to 13.9% in those receiving chemotherapy over a median follow-up of 12 months (9). The development of VTE in lung cancer patients is associated with poorer overall survival (OS), reported between 14.2 and 23.4 months for those with VTE compared to 24.4–45.8 months for those without VTE (10, 11). Furthermore, the 1-year mortality rate after a VTE diagnosis in lung cancer patients is 60.7%, establishing VTE as an independent predictor of mortality in this population (12, 13). The occurrence of VTE exposes lung cancer patients to a higher risk of death and worse prognosis, with PE being the second leading cause of death in cancer patients (14). Relevant studies suggest that lung cancer patients presenting with VTE have high early mortality rates, substantially increasing the disease burden (15, 16).

Therefore, the management of VTE is crucial in lung cancer patients. Risk assessment, anticoagulant prophylaxis, and treatment constitute the core issues of VTE management in these individuals. This complication is potentially preventable if patients at high risk for VTE are identified promptly and prophylactic anticoagulation is implemented. The situation has become more complex in the era of targeted and immunotherapy for lung cancer. Consequently, identifying risk factors in real-world settings to facilitate effective prevention holds significant clinical value and practical importance.

In this study, we enrolled 180 lung cancer patients admitted to the Fourth Affiliated Hospital of Anhui Medical University between January 2018 and April 2025. We collected their clinicopathological data, laboratory test results, and treatment regimens in an attempt to identify independent risk factors for VTE in patients with lung cancer.

## 2 Materials and methods

### 2.1 Study population

This retrospective study enrolled 180 patients with lung cancer who were admitted to the Fourth Affiliated Hospital of Anhui Medical University between January 2018 and April 2025.

The inclusion criteria were as follows: (1) aged ≥ 18 years; (2) histologically or cytologically confirmed diagnosis of lung cancer; (3) complete medical records available; and (4) available imaging findings to confirm VTE status (either positive or negative).

The exclusion criteria comprised: (1) a history of other malignant tumors; (2) a history of surgery, chemotherapy, or radiotherapy for conditions other than lung cancer within the preceding 3 months; (3) occurrence of severe infection or trauma within the last 6 weeks; (4) a previous history of thromboembolic disease; (5) use of anticoagulant or antiplatelet therapy within the 3 months before study enrollment; (6) incomplete clinical inpatient data; and (7) presence of severe hepatic failure, renal failure, or other organ dysfunction.

Patients with incomplete clinical data were excluded from the analysis; therefore, no imputation procedure was performed.

Sample size justification: This is a single-center retrospective exploratory study. The sample size was determined by the number of consecutive eligible patients admitted during the study period (January 2018 to April 2025), yielding a final cohort of 180 patients (59 VTE cases and 121 controls). For the multivariable logistic regression analysis, the events-per-variable (EPV) criterion was used to assess the adequacy of the sample size. With 59 VTE events and a maximum of 3 variables retained in the final model, the EPV was approximately 19.7, which exceeds the commonly recommended threshold of 10, indicating sufficient statistical power for the regression analyses. For subgroup analyses (e.g., embolism outside the lower extremities, *n* = 16), the EPV was below 10; therefore, only univariate analyses were performed, and these results are reported as exploratory. For machine learning models, the sample size is limited; accordingly, these analyses are strictly exploratory and hypothesis-generating, and we employed 10-fold cross-validation and bootstrap optimism-corrected internal validation (500 resamples) to mitigate overfitting and provide more stable performance estimates. The lack of a prospective power calculation is acknowledged as a limitation, and future external validation in larger cohorts is warranted.”

This study was conducted in accordance with the Declaration of Helsinki and was approved by the Ethics Committee of the Fourth Affiliated Hospital of Anhui Medical University (Approval No. KYXM-202510-001). Written informed consent was obtained from all participants.

This study was reported in accordance with the STROBE guidelines (Supplementary Figure S1).

### 2.2 Data collection

Patient baseline data were retrospectively collected through the electronic medical record system. This included age, sex, marital status, smoking history, educational level, body mass index (BMI), past medical history, and ECOG PS score. Laboratory parameters were continuously monitored, encompassing tumor markers, complete blood count, coagulation profile, hepatic and renal function, and electrolytes. All treatment strategies administered from the initial diagnosis of lung cancer until the last follow-up or death were also recorded in detail.

#### 2.2.1 Temporal relationship between D-dimer measurement and VTE diagnosis

To establish the temporal precedence of D-dimer as a potential predictive biomarker, we reviewed the electronic medical records to determine the date and clinical context of each D-dimer measurement relative to VTE diagnosis. In this cohort, D-dimer levels were measured as part of routine clinical monitoring (e.g., at hospital admission or prior to chemotherapy initiation) and preceded the diagnosis of VTE. This temporal sequence—biomarker assessment prior to the thrombotic event—supports the interpretation of D-dimer as a predictive rather than merely a diagnostic or reactive biomarker.

### 2.3 Measures for VTE assessment

The diagnosis of PE was based on computed tomographic pulmonary angiography (CTPA) or contrast-enhanced CT. The radiological criterion was a low-density filling defect within the pulmonary artery. The diagnosis of DVT primarily relied on compression duplex ultrasonography. The examined sites included the deep veins of the lower extremities (e.g., external iliac, common femoral, superficial femoral, popliteal, posterior tibial, and muscular veins), and a minority of cases involved upper extremity veins (subclavian, axillary, and brachial veins) or the external jugular vein.

### 2.4 Statistical methods

Statistical analyses were performed using SPSS software (version 27.0) and R software (version 4.6.1). The normality of continuous variables was assessed using the Shapiro-Wilk test. Accordingly, continuous variables are presented as medians with interquartile ranges (IQR), and comparisons between groups were made using the Mann-Whitney U test. Categorical variables are presented as counts with percentages [n (%)], and group comparisons were conducted using the Chi-square test or Fisher’s exact test, as appropriate.

For univariate logistic regression, continuous variables were analyzed as continuous measures (e.g., D-dimer per 1 μg/mL increase), and categorical variables were dichotomized based on clinically meaningful cutoffs or as present/absent. Variables with *p* < 0.1 in univariate analysis were entered into multivariable logistic regression. Given the limited number of events (*n* = 59), model complexity was restricted to maintain an appropriate events-per-variable ratio. Multicollinearity was assessed using variance inflation factor (VIF > 5 indicated potential collinearity). Backward stepwise selection was applied for exploratory model refinement.

For missing data, variables with more than 20% missingness were excluded (Supplementary Table S1). For variables with low-to-moderate missingness, complete-case analyses were performed. No multiple imputation was applied due to the relatively small sample size and the risk of introducing additional model uncertainty.

All tests were two-sided, and *p* < 0.05 was considered statistically significant.

### 2.5 Machine learning model development

To complement conventional regression analysis, four machine learning algorithms—logistic regression with L2 regularization (Ridge-LR), LASSO, random forest (RF), and extreme gradient boosting (XGBoost)—were applied as exploratory analytical tools to investigate potential non-linear relationships among variables associated with VTE.

The dataset was randomly split into a training set (70%, *n* = 126) and an independent test set (30%, *n* = 54). Ten-fold cross-validation was performed for hyperparameter tuning within the training set, and model stability was further assessed via bootstrap optimism-corrected validation. Given the limited sample size, these models were not intended for clinical deployment but rather for hypothesis-generating analysis.

Model performance was assessed using the area under the receiver operating characteristic curve (AUC), sensitivity, specificity, and calibration metrics, including calibration slope, intercept, and Brier score.

For the XGBoost model, the classification threshold was optimized using Youden’s index. Feature importance was estimated using the gain-based method; however, given the instability of tree-based models in small datasets, these importance measures should be interpreted with caution.

All analyses were performed using R software (version 4.6.1) with the caret and xgboost packages.

## 3 Results

### 3.1 Patient characteristics

A total of 180 patients with lung cancer were included in this study, including 59 patients in the VTE group and 121 patients in the control group. Comparisons of baseline clinical characteristics are summarized in Table 1.

**TABLE 1:** Comparison of baseline characteristics and treatment strategies between groups in lung cancer patients.

Variable	Thrombosis group (*n* = 59)	Control group (*n* = 121)	*Z*/χ^2^/*t*	*p*-value
Age (years)			4.820	0.028*
<60 years	6 (10.2%)	29 (24.0%)		
≥ 60 years	53 (89.8%)	92 (76.0%)		
Sex			1.017	0.313
Male	36 (61.0%)	83 (68.6%)		
Female	23 (39.0%)	38 (31.4%)		
Bedridden status			5.043	0.025*
Ambulatory (mobile)	48 (81.4%)	112 (92.6%)		
Bedridden	11 (18.6%)	9 (7.4%)		
Education level			6.715	0.035*
Illiterate	29 (49.2%)	39 (32.2%)		
Junior high school or below	23 (39.0%)	72 (59.5%)		
Above junior high school	7 (11.9%)	10 (8.3%)		
Smoking history			0.028	0.868
No	46 (78.0%)	93 (76.9%)		
Yes	13 (22.0%)	28 (23.1%)		
History of hypertension			1.231	0.267
No	34 (57.6%)	80 (66.1%)		
Yes	25 (42.4%)	41 (33.9%)		
History of diabetes mellitus			0.227	0.634
No	54 (91.5%)	108 (89.3%)		
Yes	5 (8.5%)	13 (10.7%)		
History of cerebral infarction			0.019	0.892
No	54 (91.5%)	110 (90.9%)		
Yes	5 (8.5%)	11 (9.1%)		
Use of calcium channel blockers			0.159	0.690
No	52 (88.1%)	109 (90.1%)		
Yes	7 (11.9%)	12 (9.9%)		
Treatment modality			1.244	0.265
Targeted therapy				
No	43 (72.9%)	85 (70.2%)		
Yes	16 (27.1%)	36 (29.8%)		
Immunotherapy			4.075	0.044*
No	41 (69.5%)	65 (53.7%)		
Yes	18 (30.5%)	56 (46.3%)		
Anti-angiogenic therapy			9.704	0.002*
No	48 (81.4%)	63 (52.1%)		
Yes	11 (18.6%)	58 (47.9%)		
Chemotherapy cycle stratification			21.742	< 0.001*
No chemotherapy	23 (39.0%)	12 (9.9%)		
1–2 cycles	14 (23.7%)	44 (36.4%)		
3–4 cycles	4 (6.8%)	16 (13.2%)		
≥ 5 cycles	18 (30.5%)	49 (40.5%)		
M stage (metastasis)			0.317	0.573
M0	26 (44.1%)	48 (39.7%)		
M1	33 (55.9%)	73 (60.3%)		
ECOG performance status			0.771	0.380
0–1	23 (39.0%)	51 (42.1%)		
2	32 (54.2%)	54 (44.6%)		
3–4	4 (6.8%)	16 (13.2%)		

Bedridden status was defined as being confined to bed for > 50% of waking hours during the hospitalization period prior to VTE assessment. Data are presented as n (%). The χ^2^ test was used for categorical variables.

*P < 0.05 indicated statistical significance.

Compared with the control group, patients with VTE were older ( ≥ 60 years: 89.8% vs. 76.0%, *p* = 0.028) and had lower educational levels (*p* = 0.035). A higher proportion of VTE patients were bedridden (18.6% vs. 7.4%, *p* = 0.025). Regarding treatment modalities, patients in the VTE group were less likely to have received immunotherapy (30.5% vs. 46.3%, *p* = 0.044), anti-angiogenic therapy (18.6% vs. 47.9%, *p* = 0.002), or chemotherapy across all cycle strata (all *p <* 0.001), with the most pronounced difference observed in the no-chemotherapy subgroup (39.0% vs. 9.9%). No significant differences were observed between the two groups regarding sex, smoking history, hypertension, diabetes mellitus, history of cerebral infarction, calcium channel blocker use, targeted therapy, M stage, or ECOG performance status (all *p >* 0.05), although a higher proportion of VTE patients had an ECOG score of 2 (54.2% vs. 44.6%).

### 3.2 Laboratory and coagulation profiles associated with VTE

Laboratory findings are presented in Table 2. Compared with the control group, patients with VTE showed significantly higher levels of carbohydrate antigen 724 (CA724), ProGRP, and D-dimer (all *p <* 0.05), as well as lower levels of total protein, albumin, and calcium (all *p <* 0.05). No significant differences were observed in other laboratory parameters including white blood cell count, hemoglobin, platelet count, INR, or AST (all *p >* 0.05). Notably, CYFRA 21-1 *(p* = 0.089) and prothrombin time (*p* = 0.071) showed borderline associations, warranting cautious interpretation.

**TABLE 2:** Laboratory findings in lung cancer patients.

Variable	Thrombosis group (*n* = 59)	Control group (*n* = 121)	*Z*/χ^2^/*t*	*p*-value
Squamous cell carcinoma antigen (SCC-Ag) (ng/mL)	1.87 (1.16,3.66)	1.87 (1.16,3.66)	−0.172	0.863
Cytokeratin 19 fragment (CYFRA 21-1) (ng/mL)	5.39 (2.77,11.34)	4.07 (2.77,11.34)	−1.699	0.089
Carbohydrate antigen 724 (CA724)	4.54 (1.63,9.84)	2.49 (1.63,9.84)	−2.894	0.004*
Neuron-specific enolase (NSE) (ng/mL)	19.30 (13.51,28.85)	19.50 (13.51,28.85)	−0.434	0.664
ProGRP	72.84 (44.15,98.70)	56.66 (44.15,98.70)	−2.818	0.005*
Carcinoembryonic antigen (CEA)	6.02 (2.73,33.03)	5.12 (2.73,33.03)	−1.230	0.219
White blood cell count ( × 10^9^/L)	6.32 (5.07,8.12)	6.20 (5.07,8.12)	−1.470	0.141
Red blood cell count ( × 10^12^/L)	3.73 (3.50,4.23)	3.89 (3.50,4.23)	-0.859	0.390
Hemoglobin (g/L)	112.00 (102.00,128.00)	117.00 (102.00,128.00)	−1.146	0.252
Platelet count ( × 10^9^/L)	183.00 (127.25,245.75)	179.00 (127.25,245.75)	−0.302	0.763
Prothrombin time (PT) (s)	11.80 (10.80,13.00)	11.60 (10.80,13.00)	−1.806	0.071
International normalized ratio (INR)	0.99 (0.91,1.13)	0.98 (0.91,1.13)	−0.706	0.480
Fibrinogen (g/L)	4.01 (3.13,5.22)	4.02 (3.13,5.22)	−0.375	0.708
D-dimer	3.01 (0.54,3.81)	1.01 (0.54,3.81)	−4.335	< 0.001*
Alanine aminotransferase (ALT) (U/L)	18.00 (13.00,28.00)	18.00 (13.00,28.00)	−1.037	0.300
Aspartate aminotransferase (AST) (U/L)	26.00 (19.25,31.75)	23.00 (19.25,31.75)	-1.644	0.100
Total bilirubin (μmol/L)	10.00 (8.00,14.50)	11.00 (8.00,14.50)	−1.569	0.117
Total protein (g/L)	65.00 (62.90,72.90)	67.40 (62.90,72.90)	−2.406	0.016*
Albumin (g/L)	37.40 (34.93,42.03)	39.40 (34.93,42.03)	−2.443	0.015*
Creatinine (μmol/L)	68.00 (55.45,86.85)	66.00 (55.45,86.85)	−0.404	0.686
Potassium (mmol/L)	4.10 (3.90,4.60)	4.00 (3.90,4.60)	−1.610	0.107
Sodium (mmol/L)	139.90 (138.00,142.00)	140.10 (138.00,142.00)	−0.504	0.614
Chloride (mmol/L)	104.00 (102.00,108.00)	104.00 (102.00,108.00)	0.971	0.331
Calcium (mmol/L)	2.21 (2.17,2.33)	2.25 (2.17,2.33)	−2.062	0.039*
Anion gap (mmol/L)	10.20 (8.90,13.70)	10.90 (8.90,13.70)	−0.791	0.429
Lactate dehydrogenase (LDH) (U/L)	239.00 (187.00,300.50)	219.00 (187.00,300.50)	−1.598	0.110

*Indicates statistical significance (*P* < 0.05).

### 3.3 Independent risk factors for VTE

Variables with *p <* 0.1 in univariate analysis were entered into multivariable logistic regression analysis (Supplementary Table S2). Prior to multivariable modeling, collinearity among candidate variables was assessed using variance inflation factor (VIF). All VIF values were below 5 (range: 1.058–1.936), indicating no significant multicollinearity (Table 3). All eight candidate variables were simultaneously entered into the multivariable model, and backward stepwise selection (likelihood ratio method) was applied with an entry criterion of *p* < 0.05 and a removal criterion of *p**** >*** 0.10.)

**TABLE 3:** Collinearity diagnostics of variables in the multivariable logistic regression model.

Variable	VIF	Tolerance
Age	1.058	0.945
Bedridden status	1.117	0.895
Chemotherapy cycle stratification	1.174	0.852
Immunotherapy	1.072	0.932
Anti-angiogenic therapy	1.098	0.911
D-dimer	1.059	0.944
Total protein	1.913	0.523

VIF < 5 indicates no significant multicollinearity. All variables met the criterion, with VIF values ranging from 1.058 to 1.936.

Univariate analysis results are presented in Table 4. Age ≥ 60 years (OR = 3.07, 95% CI: 1.06–8.92, *p* = 0.039), bedridden status (OR = 2.86, 95% CI: 1.08–7.56, *p* = 0.034), and immunotherapy (OR = 1.96, 95% CI: 1.02–3.80, *p* = 0.045) were positively associated with VTE occurrence. Chemotherapy (1–2 cycles vs. no chemotherapy) was inversely associated with VTE in univariate analysis (OR = 0.19, 95% CI: 0.08–0.46, *p**** <*** 0.001), as was anti-angiogenic therapy (OR = 0.32, 95% CI: 0.15–0.66, *p* = 0.002). Higher D-dimer levels (per 1 μg/mL increase) were associated with increased VTE risk (OR = 1.11, 95% CI: 1.04–1.19, *p* = 0.003), whereas higher total protein (OR = 0.95, 95% CI: 0.91–0.99, *p* = 0.020) and albumin (OR = 0.94, 95% CI: 0.89–0.99, *p* = 0.027) were inversely associated.

**TABLE 4:** Univariate logistic regression analysis of factors associated with VTE in lung cancer patients.

Variable	β value	OR(95% CI)	*p*-value
Age ( ≥ 60 vs. < 60 years)	1.123	3.07 (1.06–8.92)	0.039
Bedridden status (Yes vs. No)	1.050	2.86 (1.08–7.56)	0.034
Chemotherapy (1–2 cycles vs. none)	−1.661	0.19 (0.08–0.46)	< 0.001
Anti-angiogenic therapy (Yes vs. No)	−1.139	0.32 (0.15–0.66)	0.002
Immunotherapy (Yes vs. No)	0.673	1.96 (1.02–3.80)	0.045
D-dimer (per 1 μg/mL increase)	0.104	1.11 (1.04–1.19)	0.003
Total protein (per 1 g/L increase)	-0.051	0.95 (0.91–0.99)	0.020
Albumin (per 1 g/L increase)	−0.062	0.94 (0.89–0.99)	0.027

After multivariable adjustment using backward stepwise selection, three variables remained independently associated with VTE (Table 5). Age ≥ 60 years remained an independent risk factor (OR = 3.073, 95% CI: 1.059–8.916, *p* = 0.039). Compared with no chemotherapy, chemotherapy (1–2 cycles) was independently associated with increased VTE risk (OR = 2.858, 95% CI: 1.081–7.555, *p* = 0.034). D-dimer (per 1 μg/mL increase) also remained independently associated with VTE (OR = 1.084, 95% CI: 1.012–1.160, *p* = 0.021). The model showed good fit (Hosmer-Lemeshow χ^2^ = 7.310, df = 8, *p* = 0.504; Nagelkerke *R*^2^ = 0.287).

**TABLE 5:** Multivariate logistic regression analysis of factors associated with VTE in lung cancer patients.

Variable	β value	OR(95% CI)	*p*-value
Age ( ≥ 60 vs. < 60 years)	1.123	3.073(1.059–8.916)	0.039
Chemotherapy (1–2 cycles vs. none)	1.050	2.858(1.081–7.555)	0.034
D-dimer (per 1 μg/mL increase)	0.081	1.084(1.012–1.160)	0.021

(A) Model specification: Backward stepwise (likelihood-ratio) selection starting with all variables with *p* < 0.1 in univariate analysis (age, bedridden status, chemotherapy, anti-angiogenic therapy, immunotherapy, D-dimer, total protein, albumin). Removal criterion: *p* > 0.10. Final model shown above. Hosmer-Lemeshow χ^2^ = 7.310, df = 8, *p* = 0.504; Nagelkerke *R*^2^ = 0.287. (B) As detailed in Methods 2.2.1, D-dimer levels were measured as part of routine clinical monitoring prior to VTE diagnosis, establishing temporal precedence. The association therefore reflects a predictive relationship rather than reverse causality from established thrombosis. However, given the retrospective design, prospective validation is warranted.

Notably, the direction of the association for chemotherapy (1–2 cycles vs. no chemotherapy) reversed from univariate analysis (OR = 0.19, suggesting an inverse association) to multivariable analysis (OR = 2.858, indicating elevated risk). This reversal suggests that the univariate association was confounded by other covariates—particularly age and D-dimer—and underscores the importance of multivariable adjustment in interpreting treatment-related associations.

### 3.4 Clinical characteristics according to embolic site

To further evaluate factors associated with embolic location, subgroup analyses were performed in patients with VTE. Patients were stratified into a lower-extremity DVT group (*n* = 43) and an embolism-at-other-sites group (including PE, upper-extremity DVT, and combined PE with lower-extremity DVT; *n* = 16). As shown in Table 6, compared with patients with isolated lower-extremity DVT, those with embolism at other sites were more likely to be male (87.5% vs. 51.2%, *p* = 0.011), had poorer ECOG performance status (PS 5: 75.0% vs. 46.5%, *p* = 0.046), and were more likely to have received immunotherapy (54.5% vs. 16.2%, *p* = 0.002). No significant differences were observed in other baseline characteristics or treatment modalities (all *p >* 0.05). Laboratory parameters also showed no significant differences between the two groups (Table 7, all *p >* 0.05).

**TABLE 6:** Comparison of baseline characteristics and treatment strategies between lung cancer patients with lower extremity DVT and embolism at other sites.

Variable	Lower extremity DVT group (*n* = 43)	Embolism at other sites group (*n* = 16)	*Z*/χ^2^/*t*	*p*-value
Age (years)			−0.157	0.875
< 60 years	2 (4.7%)	4 (25.0%)		
≥ 60 years	41 (95.3%)	12 (75.0%)		
Sex			6.473	0.011*
Male	22 (51.2%)	14 (87.5%)		
Female	21 (48.8%)	2 (12.5%)		
Bedridden status			1.722	0.189
No	–	–		
Yes	–	–		
Education level			1.497	0.221
Illiterate	23 (53.5%)	6 (37.5%)		
Junior high school or below	16 (37.2%)	7 (43.8%)		
Above junior high school	4 (9.3%)	3 (18.8%)		
Smoking history			–	0.184
No	32 (74.4%)	14 (87.5%)		
Yes	11 (25.6%)	2 (12.5%)		
History of hypertension			1.112	0.292
No	23 (53.5%)	11 (68.8%)		
Yes	20 (46.5%)	5 (31.3%)		
History of diabetes mellitus			–	0.125
No	39 (90.7%)	15 (93.8%)		
Yes	4 (9.3%)	1 (6.3%)		
History of cerebral infarction			–	0.597
No	39 (90.7%)	15 (93.8%)		
Yes	4 (9.3%)	1 (6.3%)		
Use of calcium channel blockers			–	0.906
No	37 (86.0%)	15 (93.8%)		
Yes	6 (14.0%)	1 (6.3%)		
Treatment modality				
Targeted therapy			0.392	0.531
Yes	9 (24.3%)	7 (31.8%)		
No	28 (75.7%)	15 (68.2%)		
Immunotherapy			9.560	0.002*
Yes	6 (16.2%)	12 (54.5%)		
No	31 (83.8%)	10 (45.5%)		
Anti-angiogenic therapy			0.386	0.535
Yes	6 (16.2%)	5 (22.7%)		
No	31 (83.8%)	17 (77.3%)		
Chemotherapy cycle stratification			2.769	0.429
No chemotherapy	17 (45.9%)	6 (27.3%)		
1–2 cycles	9 (24.3%)	5 (22.7%)		
3–4 cycles	2 (5.4%)	2 (9.1%)		
≥ 5 cycles	9 (24.3%)	9 (40.9%)		
M stage			1.463	0.226
M0	21 (48.8%)	5 (31.3%)		
M1	22 (51.2%)	11 (68.8%)		
ECOG performance status			3.987	0.046*
0–1	20 (46.5%)	3 (18.8%)		
2	—	—		
3–4	3 (7.0%)	1 (6.3%)		

*Indicates statistical significance (*P* < 0.05).

**TABLE 7:** Comparison of laboratory findings between lung cancer patients with lower extremity DVT and embolism at other sites.

Lower extremity variable	DVT Group (*n* = 43)	Embolism at Other Sites Group (*n* = 16)	*Z*/χ^2^/*t*	*p*-value
SCC-Ag (ng/mL)	1.69 (0.97,5.81)	2.09 (1.08,3.40)	−0.212	0.832
CYFRA 21-1 (ng/mL)	6.36 (3.19,17.20)	3.94 (2.73,8.76)	−1.215	0.224
CA724	3.69 (2,08,14.46)	7.94 (1.98,15.97)	−0.361	0.718
NSE (ng/mL)	20.60 (13.75,30.12)	16.39 (12.60,23.16)	−1.027	0.304
ProGRP	83.40 (49.65,182.11)	72.12 (59.12,109.85)	−0.071	0.944
CEA	6.70 (2.99,88.47)	4.66 (2.24,73.98)	−0.988	0.323
WBC ( × 10^9^/L)	6.32 (5.12,9.04)	6.32 (5.16,9.31)	−0.204	0.839
RBC ( × 10^12^/L)	3.60 (3.34,4.18)	3.83 (3.53,4.22)	−0.972	0.331
Hemoglobin (g/L)	111.00 (99.00,123.50)	114.50 (103.25,126.50)	−0.486	0.627
Platelet ( × 10^9^/L)	183.00 (118.00,257.50)	204.00 (137.00,268.75)	−0.776	0.438
PT (s)	11.70 (11.05,13.60)	11.95 (10.83,13.15)	−0.463	0.644
INR	1.00 (0.95,1.19)	0.96 (0.90,1.05)	−1.522	0.128
Fibrinogen (g/L)	4.01 (3.04,5.25)	4.26 (3.29,4.90)	−0.698	0.485
D-dimer	3.44 (0.73,8.93)	2.49 (1.26,8.19)	−0.024	0.981
ALT (U/L)	18.00 (12.00,41.50)	18.50 (15.75,36.00)	−1.051	0.293
AST (U/L)	26.00 (19.00,34.00)	27.00 (21.75,38.25)	−0.855	0.393
Total bilirubin (μmol/L)	11.00 (7.00,14.15)	9.50 (6.58.11.00)	−1.633	0.102
Total protein (g/L)	64.10 (59.60,69.20)	65.85 (61.80,70.20)	−0.604	0.546
Albumin (g/L)	35.60 (33.15,40.95)	38.75 (33.45,42.15)	−0.917	0.359
Creatinine (μmol/L)	68.00 (53.75,91.50)	70.50 (56.75,81.25)	−0.196	0.845
Potassium (mmol/L)	4.00 (3.82,4.41)	4.18 (3.89,4.53)	−0.753	0.451
Sodium (mmol/L)	139.40 (136.00,141.90)	140.10 (138.53,141.45)	−0.047	0.962
Chloride (mmol/L)	104.00 (101.50,107.50)	104.00 (100.75,107.00)	−0.079	0.937
Calcium (mmol/L)	2.20 (2.13,2.29)	2.24 (2.09,2.30)	−0.690	0.490
Anion gap (mmol/L)	10.50 (9.00,13.15)	10.00 (8.35,12.40)	−0.784	0.433
LDH (U/L)	269.00 (191.50,300.50)	239.00 (192.75,300.50)	−0.347	0.729

### 3.5 Factors Associated with embolism outside the lower extremities

Univariate logistic regression was performed to identify factors associated with embolism outside the lower extremities (Table 8). Immunotherapy was significantly associated with embolism outside the lower extremities (OR = 6.200, 95% CI: 1.846–20.829, *p* = 0.003), whereas age (*p* = 0.137) and sex *(p* = 0.159) were not statistically significant.

**TABLE 8:** Univariate logistic regression analysis of factors influencing embolism type (lower extremity DVT Group vs. embolism at other sites group).

Variable	β value	OR(95% CI)	*p*-value
Age ( ≥ 60 years vs. < 60 years)	−1.818	0.257 (0.043,1.540)	0.137
Sex (Male vs. Female)	2.268	0.441 (0.141,1.379)	0.159
Immunotherapy (Yes vs. No)	1.825	6.200 (1.846,20.829)	0.003*

Due to the extremely small sample size (*n* = 16 events in the “embolism at other sites” group), these univariate odds ratios are statistically unstable with wide confidence intervals. These findings should be interpreted as exploratory and hypothesis-generating only; they are not suitable for clinical decision-making and require validation in larger cohorts. Multivariable adjustment was not performed because the events-per-variable ratio was below the recommended threshold of 10.

*Indicates statistical significance (*P* < 0.05).

Owing to the limited sample size—only 16 cases in the embolism-at-other-sites group—multivariable adjustment was not performed, as the events-per-variable ratio fell below the recommended threshold of 10 (allowing adjustment for only one variable). These findings should be considered exploratory and should be validated in larger prospective cohorts.

### 3.6 Machine learning-based exploratory analysis

To complement conventional regression analysis, four machine learning models—Ridge-LR, LASSO, random forest (RF), and XGBoost—were developed as exploratory tools for VTE risk prediction. Given the limited sample size (*n* = 180, with 59 VTE events), these analyses were strictly exploratory and intended for hypothesis generation rather than confirmatory prediction.

#### 3.6.1 Model performance and calibration

**FIGURE 1:**
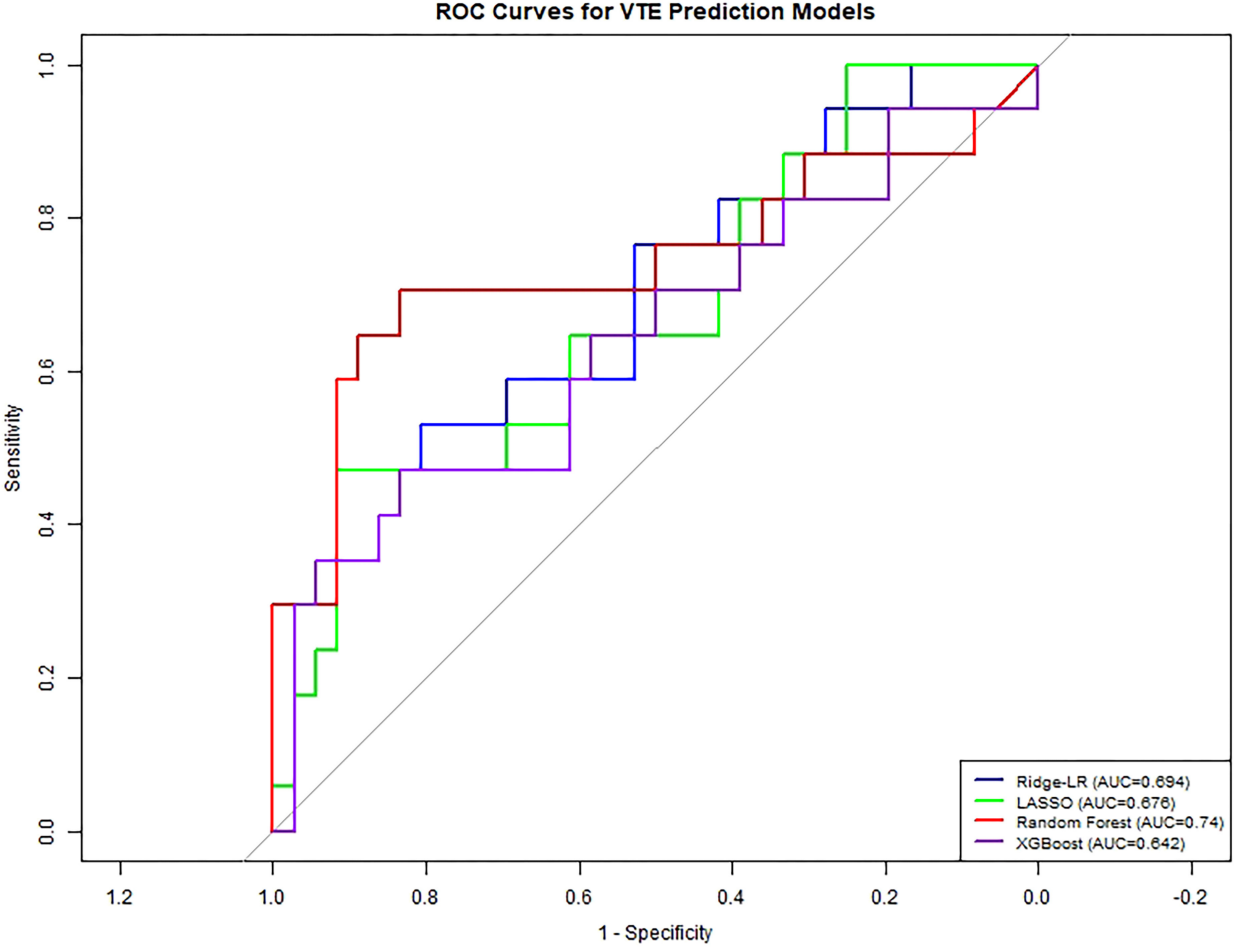
Receiver operating characteristic (ROC) curves for VTE prediction models.

Model performance metrics on the test set are summarized in Figure 1 and Table 9. RF achieved the highest AUC of 0.740, with a sensitivity of 0.706 and specificity of 0.833. Ridge-LR showed an AUC of 0.694 (sensitivity 0.471, specificity 0.917), and LASSO achieved an AUC of 0.676 (sensitivity 0.471, specificity 0.917). XGBoost showed the lowest AUC of 0.642 (sensitivity 0.471, specificity 0.833).

**TABLE 9:** Performance comparison of machine learning models on the test set.

Metric	Ridge-LR	LASSO	Random Forest	XGBoost
AUC	0.694	0.676	0.740	0.642
Sensitivity	0.471	0.471	0.706	0.471
Specificity	0.917	0.917	0.833	0.833
Brier Score	0.196	0.200	0.180	0.221
Calibration Slope	0.664	0.375	0.618	0.348
Calibration Intercept	−0.346	−0.576	−0.160	−0.470
Hosmer-Lemeshow χ^2^ (*p*-value)	8.963 (0.345)	15.297 (0.054)	24.530 (0.002)	24.476 (0.002)

AUC, Area Under the Curve. Brier score ranges from 0 to 1, with lower values indicating better overall performance. A perfectly calibrated model has calibration slope = 1.0 and intercept = 0. Hosmer-Lemeshow test: χ^2^ with df = 8; *p* > 0.05 indicates good model fit.

**FIGURE 2:**
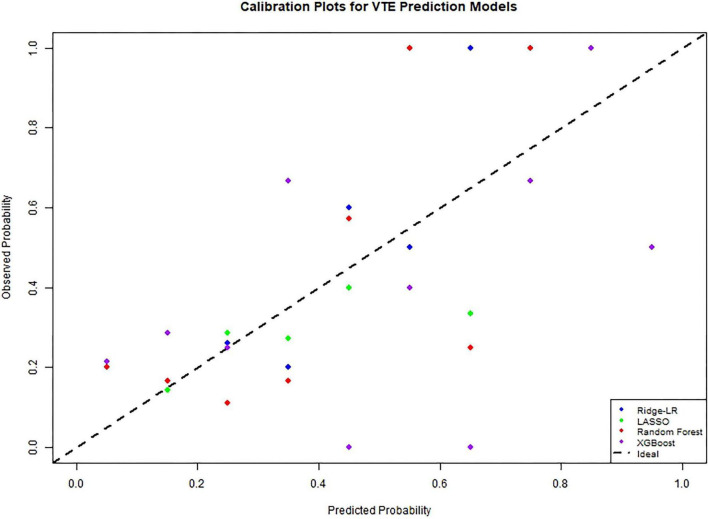
Calibration plots for VTE prediction models.

Calibration assessment revealed that all models exhibited miscalibration to varying degrees (Figure 2 and Table 9). Ridge-LR showed the best calibration performance (slope: 0.664, intercept: −0.346), followed by RF (slope: 0.618, intercept: −0.160). LASSO and XGBoost showed poorer calibration, with slopes of 0.375 and 0.348, respectively, indicating systematic overestimation of risk probabilities. Brier scores ranged from 0.180 (RF) to 0.221 (XGBoost), with lower scores indicating better overall performance.

To obtain more stable performance estimates, 10-fold cross-validation (repeated 10 times) and bootstrap optimism-corrected validation (500 resamples) were performed (Tables 10, 11). Cross-validation yielded mean AUCs of 0.715 (Ridge-LR), 0.681 (LASSO), 0.749 (RF), and 0.707 (XGBoost), with wide confidence intervals reflecting the limited sample size. The optimism-corrected bootstrap AUCs were 0.713 (Ridge-LR), 0.725 (LASSO), 0.745 (RF), and 0.868 (XGBoost). These corrected estimates were largely consistent with the cross-validation and test-set results for Ridge-LR, LASSO, and RF, confirming their stability. In contrast, XGBoost showed a high apparent AUC of 0.976 with an optimism of 0.108, indicating substantial overfitting that was only partially corrected; its relatively low test-set performance (AUC 0.642) further cautions against its use in this small cohort.

**TABLE 10:** Cross-validation performance (10-fold, repeated 10 times).

Model	AUC (Mean)	AUC (SD)	95% CI
Ridge-LR	0.715	0.121	0.458–0.917
LASSO	0.681	0.115	0.453–0.871
Random Forest	0.749	0.113	0.538–0.983
XGBoost	0.707	0.117	0.507–0.946

**TABLE 11:** Bootstrap optimism-corrected internal validation (500 resamples).

Model	Apparent_AUC	Mean_Optimism	Corrected_AUC
Ridge-LR	0.761	0.047	0.713
LASSO	0.779	0.054	0.725
Random Forest	0.738	−0.006	0.745
XGBoost	0.976	0.108	0.868

The Hosmer-Lemeshow test yielded χ^2^ values of 8.963 (*p* = 0.345) for Ridge-LR, 15.297 (*p* = 0.054) for LASSO, 24.530 (*p* = 0.002) for RF, and 24.476 (*p* = 0.002) for XGBoost (Table 9). These results indicate acceptable calibration only for Ridge-LR, while LASSO showed borderline lack of fit, and both tree-based models demonstrated significant lack of fit.

#### 3.6.2 Threshold optimization analysis

Threshold optimization using Youden‘s index was performed for XGBoost (Table 12). The optimal threshold was 0.60, yielding a sensitivity of 0.353 and specificity of 0.917, compared with a sensitivity of 0.471 and specificity of 0.833 at the default threshold of 0.50.

**TABLE 12:** XGBoost threshold optimization.

Threshold	Sensitivity	Specificity
0.50	0.471	0.833
0.55	0.353	0.861
0.60	0.353	0.917
0.65	0.353	0.917
0.70	0.353	0.944
0.75	0.294	0.944
0.80	0.235	0.972

The optimal threshold determined by Youden’s index was 0.60 (sensitivity 0.353, specificity 0.917).

#### 3.6.3 Feature importance analysis

Feature importance analysis using the XGBoost gain method identified D-dimer (41.3%) and total protein (34.7%) as the two most important predictors, collectively accounting for 76.0% of the total gain (Table 13). Immunotherapy (7.7%), anti-angiogenic therapy (4.6%), and chemotherapy ≥ 5 cycles (4.4%) showed relatively lower importance.

**TABLE 13:** Top 5 feature importance rankings.

Rank	Feature	Importance (%)
1	D-dimer	41.3
2	Total protein	34.7
3	Immunotherapy	7.7
4	Anti-angiogenic therapy	4.6
5	Chemotherapy ( ≥ 5 cycles)	4.4

#### 3.6.4 Decision curve analysis

**FIGURE 3:**
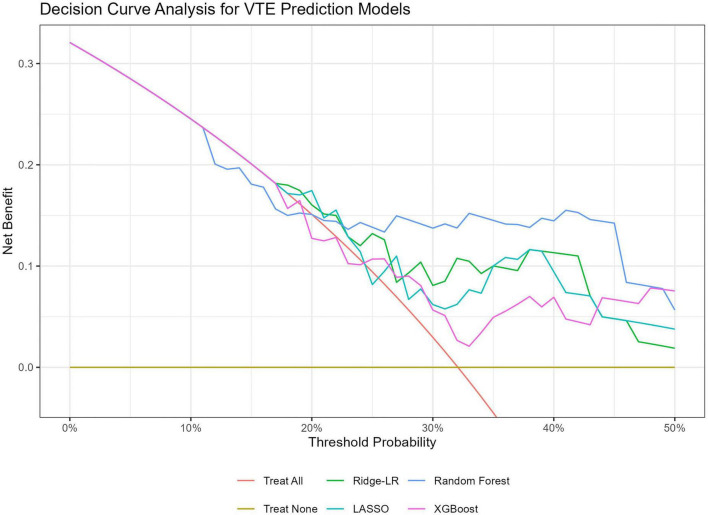
Decision curve analysis (DCA) for VTE prediction models.

Decision curve analysis was performed to evaluate the clinical utility of each model across a range of threshold probabilities (Figure 3). Among all models, LASSO demonstrated the highest net benefit across the threshold range of 10–50%, essentially equivalent to the “treat all” strategy. XGBoost also showed positive net benefit across the same threshold range, though lower than that of LASSO. Ridge-LR and random forest showed no net benefit over the “treat none” strategy across all thresholds. These findings suggest that, within the current dataset, LASSO offers the most favorable trade-off between true-positive and false-positive classifications for guiding VTE prevention decisions.

Overall, although random forest showed the highest AUC in some analyses, calibration assessment and decision curve analysis suggested limited improvement over simpler approaches. These results should be considered exploratory, and require validation in larger independent cohorts.

## 4 Discussion

In this single-center retrospective study of patients with lung cancer, we identified several clinically relevant findings regarding risk factors for venous thromboembolism (VTE) and predictive modeling.

Elevated D-dimer levels were independently associated with subsequent VTE in patients with lung cancer. A key strength of this study is the temporal sequencing, as D-dimer measurements were obtained during routine clinical monitoring before VTE diagnosis, rather than at or after the thrombotic event. This temporal design reduced the likelihood of reverse causality—a common limitation in retrospective biomarker studies—and supported its potential role in risk stratification. This finding is consistent with previous reports identifying D-dimer as a marker of cancer-associated thrombosis (17–19) and extends prior evidence by clarifying its temporal relationship with clinical outcomes.

Nevertheless, residual uncertainty remains, as subclinical thrombosis at the time of blood sampling cannot be excluded, and serial measurements were not available to assess temporal dynamics. Therefore, these findings should be considered hypothesis-generating and require prospective validation.

An inverse association between chemotherapy exposure and VTE was observed in univariate analysis, whereas multivariable adjustment suggested a positive association. This inconsistency likely reflects confounding by indication and treatment selection bias rather than a true biological effect. Patients receiving chemotherapy likely represent a clinically selected subgroup with distinct baseline characteristics, including disease severity and overall treatment strategy. Therefore, chemotherapy exposure in this study should be interpreted as a surrogate marker of clinical decision-making rather than an independent causal risk factor (20, 21).

In addition, male sex was associated with embolic events occurring outside the lower extremities, including pulmonary embolism, suggesting potential sex-related heterogeneity in the clinical phenotype of VTE in lung cancer patients. However, biological mechanisms remain unclear, and current evidence from registry-based studies suggests that sex differences in cancer-associated thrombosis exist but remain inconsistently characterized across tumor types (22).

From a methodological perspective, machine learning models did not demonstrate consistent improvement over conventional logistic regression in this dataset. Although some models showed moderate discrimination, performance was unstable across calibration and validation metrics. These findings suggest limited incremental value of complex machine learning approaches in small single-center datasets and are consistent with prior literature indicating that traditional regression models often remain competitive in such settings (23, 24).

This study has several limitations. First, its retrospective single-center design limits generalizability and introduces potential selection bias. Second, chemotherapy exposure could not be further stratified by regimen, cumulative dose, or concurrent therapies, resulting in residual confounding. Third, the relatively small number of events limited the stability of complex machine learning models. Finally, external validation was not performed, which may have led to optimistic performance estimates. In addition, no correction for multiple comparisons was applied in the subgroup or model comparisons, which may increase the risk of false-positive findings; therefore, these results should be interpreted as exploratory and hypothesis-generating.

In conclusion, D-dimer levels were independently associated with VTE risk in patients with lung cancer and may serve as a clinically useful biomarker for risk stratification. Machine learning approaches did not provide additional predictive benefit compared with logistic regression in this cohort.

## Data Availability

The raw data supporting the conclusions of this article will be made available by the authors, without undue reservation.
