# Supplementary material for: Clinical risk factors and machine learning for venous thromboembolism in lung cancer: an exploratory single-center study
Source: Front Med (Lausanne). 2026 Jul 31;13:1891648. doi: 10.3389/fmed.2026.1891648 (PMC13473358; doi:10.3389/fmed.2026.1891648)
Supplement: Supplementary file 1 [file Supplementary_file_1.docx]

### Supplementary Table 1.Missing Data Summary

| Variable | Valid Cases (n) | Missing Cases (n) | Missing Rate (%) | Handling Method |
| --- | --- | --- | --- | --- |
| TPA | 43 | 137 | 76.1 | Excluded |
| HSP90α | 74 | 106 | 58.9 | Excluded |
| VEGF | 75 | 105 | 58.3 | Excluded |
| High‑sensitivity troponin | 37 | 143 | 79.4 | Excluded |
| α‑hydroxybutyrate dehydrogenase | 123 | 57 | 31.7 | Excluded |
| Creatine kinase | 124 | 56 | 31.1 | Excluded |
| Procalcitonin | 26 | 154 | 85.6 | Excluded |
| BNP | 43 | 137 | 76.1 | Excluded |
| FT3 | 12 | 168 | 93.3 | Excluded |
| FT4 | 12 | 168 | 93.3 | Excluded |
| TT3 | 74 | 106 | 58.9 | Excluded |
| TT4 | 76 | 104 | 57.8 | Excluded |
| TSH | 83 | 97 | 53.9 | Excluded |
| ProGRP | 171 | 9 | 5.0 | Mean imputation |
| CEA | 175 | 5 | 2.8 | Mean imputation |
| SCC | 172 | 8 | 4.4 | Mean imputation |
| CYFRA21‑1 | 174 | 6 | 3.3 | Mean imputation |
| CA724 | 168 | 12 | 6.7 | Mean imputation |
| NSE | 173 | 7 | 3.9 | Mean imputation |
| PT | 177 | 3 | 1.7 | Mean imputation |
| INR | 177 | 3 | 1.7 | Mean imputation |
| Fibrinogen | 177 | 3 | 1.7 | Mean imputation |
| D‑dimer | 177 | 3 | 1.7 | Mean imputation |
| ALT | 173 | 7 | 3.9 | Mean imputation |
| Calcium | 179 | 1 | 0.6 | Mean imputation |
| Anion gap | 179 | 1 | 0.6 | Mean imputation |
| LDH | 150 | 30 | 16.7 | Multiple imputation |

Note:TPA, tissue polypeptide antigen; HSP90α, heat shock protein 90α; VEGF, vascular endothelial growth factor; BNP, brain natriuretic peptide; FT3, free triiodothyronine; FT4, free thyroxine; TT3, total triiodothyronine; TT4, total thyroxine; TSH, thyroid‑stimulating hormone; ProGRP, pro‑gastrin‑releasing peptide; CEA, carcinoembryonic antigen; SCC, squamous cell carcinoma antigen; CYFRA21‑1, cytokeratin 19 fragment; CA724, cancer antigen 724; NSE, neuron‑specific enolase; PT, prothrombin time; INR, international normalized ratio; ALT, alanine aminotransferase; LDH, lactate dehydrogenase.

### Supplementary Table S2. Candidate Variables with p < 0.1 in Univariable Analysis

| No. | Variable | Type | ***P-value*** |
| --- | --- | --- | --- |
| 1 | Chemotherapy cycle stratification | Categorical | <0.001 |
| 2 | D‑dimer | Continuous | <0.001 |
| 3 | Anti‑angiogenic therapy | Categorical | 0.002 |
| 4 | CA724 | Continuous | 0.004 |
| 5 | ProGRP | Continuous | 0.005 |
| 6 | Albumin | Continuous | 0.015 |
| 7 | Total protein | Continuous | 0.016 |
| 8 | Age | Categorical | 0.028 |
| 9 | Education level | Categorical (3 groups) | 0.035 |
| 10 | Calcium | Continuous | 0.039 |
| 11 | Immunotherapy | Categorical | 0.045 |
| 13 | Prothrombin time | Continuous | 0.071 |
| 14 | Cytokeratin 19 fragment | Continuous | 0.089 |
| 15 | BMI | Categorical (4 groups) | 0.091 |

Note: Variables with p < 0.1 in univariable analysis were entered into multivariable logistic regression. The full list of candidate variables (n = 15) is presented here; variable types and p‑values are clearly annotated. No. 12 is not listed as no variable met the inclusion criterion at that position in the original analysis sequence.

Supplementary Figure S1 STROBE flow diagram of patient selection.
